# Haptic Virtual Reality Training for Combined Spinal-Epidural Anesthesia in Anesthesiology Trainees: Prospective Randomized Controlled Pilot Trial

**DOI:** 10.2196/92207

**Published:** 2026-10-08

**Authors:** Huihong Xie, Weiwei Lin, Yinjie Zheng, Mingxue Lin, Xiaobin Fang, Simeng Ma, Fu-Shan Xue, Xiaochun Zheng, Danfeng Wang, Fei Gao

**Affiliations:** 1 Department of Anesthesiology, Fuzhou University Affiliated Provincial Hospital, Shengli Clinical Medical College of Fujian Medical University, Fujian Provincial Hospital, No. 134 Dongjie Street, Gulou District, Fuzhou, 350001, China, 86 13559434579; 2 Fujian Provincial Key Laboratory of Critical Care Medicine, Fujian Emergency Medical Center, Fuzhou, China

**Keywords:** virtual reality, haptic feedback, combined spinal-epidural, medical education, pilot trial, skill transfer, serious games, psychomotor skills, gamification

## Abstract

**Background:**

Combined spinal-epidural (CSE) anesthesia is a complex psychomotor skill requiring precise tactile discrimination. Traditional manikin-based training may be limited by degraded haptic fidelity and inconsistent tissue feedback. Whether skills acquired through haptic virtual reality (VR) training transfer to supervised clinical procedures remains uncertain.

**Objective:**

This study assessed the feasibility and early clinical application of CSE skills after high-fidelity haptic VR training in junior anesthesiology trainees vs traditional manikin-based training.

**Methods:**

We conducted a single-center, prospective, parallel-group, randomized, and outcome assessor-blind pilot trial. A total of 20 fifth-year anesthesiology interns with minimal neuraxial experience were randomly assigned (1:1) by an independent investigator using a card draw to a standardized 7-day curriculum with either a traditional manikin (control group [CG], n=10) or a haptic-VR simulator (simulator group [SG], n=10). SG trainees performed repeated punctures on the haptic-VR CSE Teaching Platform, whereas the CG trainees practiced the CSE procedure on a lumbar puncture manikin. Both interventions were delivered face-to-face in a simulation laboratory by experienced instructors. During training, each trainee performed 1 supervised CSE on a patient. Clinical performance was video recorded and assessed by blinded experts. The primary outcome was the 0 to 100 total clinical performance score, rated by 2 assessors. Secondary outcomes included domain-specific scores, procedural time, puncture-angle adjustments, first-attempt success rate, and adverse events within 24 hours.

**Results:**

All 20 trainees completed training and clinical assessments and were included in the intention-to-treat analysis. Overall scores were higher in the SG than in the CG (mean difference 4.50 points, 95% CI 1.85-7.15; *P*=.002). The SG had higher scores for tactile-dependent skills, including recognition by bubble compression (mean difference 1.90 points, loss of resistance 95% CI 1.14-2.66; *P*<.001) and ligamentum flavum penetration (mean difference 1.90 points, 95% CI 1.18-2.62; *P*<.001), but lower aseptic technique compliance (mean difference −1.40 points, 95% CI −2.00 to −0.80; *P*<.001). The SG trainees had a shorter time to ligamentum flavum breakthrough, fewer passive angle adjustments, and more active adjustments. The first-attempt success rate was higher but imprecisely estimated. Primary-outcome interrater reliability was excellent (average-measures intraclass correlation coefficient of 0.977). No significant adverse events occurred.

**Conclusions:**

Compared with traditional manikin training, haptic-VR training yielded higher overall clinical performance and better tactile-dependent CSE skills during the first supervised patient procedure, whereas manikin training better supported aseptic practice. By evaluating posttraining performance in patients rather than only in simulation, this study extends VR research toward early clinical skill transfer and suggests that the 2 modalities may develop complementary components of procedural competence. In practice, these findings support a blended CSE curriculum in which VR is used for repeated tactile and needle-control training and manikin-based simulation for aseptic preparation and procedural workflow; this approach should be evaluated in larger trials.

## Introduction

Combined spinal-epidural (CSE) anesthesia is widely used in anesthesiology, especially for obstetric and lower-limb orthopedic surgeries [1,2]. This technique pairs the rapid onset of spinal anesthesia with the option of epidural catheterization when prolonged analgesia is needed [3,4]. In practice, CSE is demanding for less experienced operators. Before advancing the spinal needle through the epidural needle, the operator must identify surface anatomical landmarks and recognize the epidural space through tactile feedback, most often by detecting loss of resistance (LOR) [5,6]. Although the needle-through-needle approach is commonly used, it adds technical difficulty because problems such as failed dural puncture or paresthesia may occur, particularly when the operator has limited procedural experience [5,7-9].

For many residents, CSE is traditionally learned through supervised procedures on patients [10-12]. That route remains part of training, but patient safety concerns, ethical constraints, and reduced clinical training time have made simulation-based medical education a larger part of procedural teaching [13,14]. Physical manikins are commonly used for this purpose, but their tactile feel can change with use. When a silicone or rubber model is punctured repeatedly, the needle may leave tracks in the material and create an easier route for later attempts. Trainees may then practice on resistance that no longer matches what they are expected to feel during the epidural space identification, especially when learning the LOR technique [15,16]. For CSE training, an effective simulator should do more than allow trainees to repeat the steps. It should give them similar resistance cues each time they advance the needle, including the tissue-layer resistance and LOR cues used to identify the epidural space.

Virtual reality (VR) and extended reality (XR) offer one way to build this type of practice into anesthesiology training. By linking 3D visualization with biomechanical models and haptic devices, these systems allow trainees to rehearse complex procedures under controlled conditions before working with patients [17-19]. In anesthesiology, VR-based simulation has been applied to airway management, regional anesthesia, and neuraxial procedures; studies in these areas have described improvements in technical performance, spatial understanding, and training efficiency [18-21]. Work focused on neuraxial procedures, including scoping reviews and experimental studies, also suggests that XR can reproduce several procedural elements that matter for training and guidance [17]. What remains less clear is what happens after simulator-based practice. Much of the literature still reports simulator scores, learner satisfaction, or cognitive workload [22,23], whereas only a few studies have examined VR-based training in clinical settings. Whether skills acquired in VR carry over to patient care is still uncertain [17,24].

In CSE, this question is not only theoretical. During needle advancement, trainees must detect small changes in resistance, but the same procedure also requires sterile preparation, correct sequencing of each step, and decisions about when to redirect the needle or continue. A VR program may improve the haptic part of CSE without producing the same gain in workflow or aseptic performance. Examining these domains separately can show where VR adds value in anesthesia training and where physical simulation may still be needed. In this study, the haptic-VR platform was evaluated as a supervised procedural training tool within simulation-based anesthesiology education rather than as a stand-alone patient-care intervention.

Therefore, we conducted a prospective, randomized pilot trial comparing a haptic-feedback VR platform with traditional high-fidelity manikin training. The trial had 2 main aims. First, we assessed whether puncture skills learned in VR could transfer to an early, supervised CSE procedure in an eligible patient, with the total clinical performance score as the primary outcome. Second, we examined whether VR and manikin-based training affected different parts of procedural performance in different ways.

## Methods

### Trial Design

This study was a single-center, prospective, parallel-group, randomized, and outcome assessor-blind exploratory pilot trial, with individual trainees as the unit of randomization and a 1:1 allocation ratio, designed to evaluate the feasibility and preliminary educational effects of VR-based CSE training compared with traditional manikin-based training. The trainees underwent training in a dedicated simulation laboratory, and clinical assessments were conducted in the operating room. The trial was registered with the Chinese Clinical Trial Registry (registration: ChiCTR2100043952) on March 6, 2021. Trainee enrollment began after trial registration, and the first trainee participant was enrolled on March 7, 2021. Clinical patient enrollment for the supervised procedural assessment began on March 10, 2021. The last participant was enrolled on March 31, 2021. No trainee or patient participant was enrolled before trial registration. The study protocol, including the eligibility criteria, intervention design, and outcome measures, was finalized prior to participant screening and remained unchanged throughout the study. A separate formal statistical analysis plan was not published before the trial. The trial ended as planned after completing the prespecified pilot sample. This study was reported in accordance with the CONSORT (Consolidated Standards of Reporting Trials) 2025 statement for randomized controlled trials [25]. Completed CONSORT 2025 and CONSORT-EHEALTH (Consolidated Standards of Reporting Trials of Electronic and Mobile Health Applications and Online Telehealth) checklists [26] are provided as supplementary files (Checklists 1 and 2). The intervention evaluated in this trial was a haptic-VR training simulator and did not include an AI component, such as automated diagnosis, prediction, clinical decision support, adaptive training recommendations, or algorithm-generated feedback. Therefore, the CONSORT-AI (Consolidated Standards of Reporting Trials–AI) extension was not applicable to this study. This was not an open web-based or mobile-app trial. Recruitment and written informed consent were conducted offline at the hospital, training was delivered face-to-face in a dedicated simulation laboratory, and clinical assessments were conducted in the operating room. The VR platform was used as an offline supervised training simulator and did not require public web access, user accounts, logins, online questionnaires, automated email/SMS prompts, or remote self-directed use.

### Ethical Considerations

This study was approved by the Ethics Committee of Fujian Provincial Hospital (approval K2020-12-018) and was conducted in accordance with the Declaration of Helsinki. All participating trainees and patients provided written informed consent before enrollment. Consent was obtained offline using written consent forms after trainees and patients had been informed of the study purpose, procedures, the voluntary nature of participation, privacy protection, and the planned use of deidentified research data. For patients undergoing clinical procedures, consent included permission to use deidentified procedural data for research purposes. All study data were anonymized before analysis, and video recordings were used solely for blinded expert assessment and deidentified before evaluation. No identifiable personal information was included in the analytic dataset, reported in the manuscript, or included in the supplementary materials. Participation was voluntary, and participants received no financial compensation. No images or materials in the manuscript or supplementary files contained identifiable participant information.

### Patient and Public Involvement

The patients or the public were not involved in the design, conduct, reporting, or dissemination of this study.

### Participants

#### Trainees

A total of 20 fifth-year anesthesiology interns undergoing clinical internships at Fujian Medical University with limited prior experience in neuraxial anesthesia were enrolled. Prior experience was defined as any attempt at needle manipulation in a patient, whether successful or unsuccessful. The inclusion threshold was set at fewer than 10 prior supervised neuraxial procedures to capture early learners. In practice, all enrolled participants met our operational definition of “true novices,” defined as trainees with no more than 3 prior supervised neuraxial needle-manipulation attempts. Computer, internet, or eHealth literacy was not an eligibility criterion and was not separately assessed because trainees used the VR platform only during supervised face-to-face sessions with instructor support in the simulation laboratory.

#### Patients

Twenty patients with lower-limb fractures scheduled to undergo lower-limb arthroplasty under CSE anesthesia were enrolled. All patients were screened before enrollment according to predefined inclusion and exclusion criteria to reduce patient-related variability in procedural difficulty. Inclusion criteria for the study were as follows: indication for neuraxial puncture; age 35 to 65 years, inclusive; BMI 18 to 25 kg/m²; American Society of Anesthesiologists physical status I to II; no contraindication to neuraxial anesthesia; no history of spinal deformity or spinal surgery; no skin infection at the puncture site; and provision of written informed consent. Exclusion criteria were allergy to amide local anesthetics, central nervous system disease, abnormal function of the heart, lungs, kidneys, or other major organs, lower-limb motor or sensory abnormalities, and mental disorders or communication disorders. Only patients who met all eligibility criteria were included in the clinical assessment phase.

### Randomization and Blinding

Trainees were randomly assigned in a 1:1 ratio to either the control group (CG, n=10) or the simulator group (SG, n=10) using a lottery-based (card-draw) method without blocking or stratification. An investigator, independent of participant recruitment, training delivery, clinical supervision, and outcome assessment, prepared 10 red cards and 10 yellow cards in advance and placed them in an opaque container; red cards corresponded to the CG and yellow cards corresponded to the SG. Each trainee drew 1 card at random from the container, and the allocation was revealed and recorded immediately. Group assignment was disclosed only after trainee enrollment and before the assigned training intervention began. The outcome assessors remained blinded to the trainee group assignment throughout the video-based assessment.

Before the clinical assessment, a separate randomized trainee procedure order was generated using the RAND() function in Microsoft Excel. Each trainee was assigned a random number, and trainees were sorted by these numbers to create a prespecified procedure order from 1 to 20.

All patients were screened according to predefined inclusion and exclusion criteria. Eligible patients entered the clinical assessment sequence consecutively according to the order in which their surgeries were scheduled. The trainee who performed the CSE procedure for each patient was determined by the prespecified randomized trainee order from 1 to 20. In other words, the first eligible surgical patient was treated by the trainee ranked first in the random order, the second eligible patient by the trainee ranked second, and so forth. Therefore, patients were not selectively matched to trainees based on training group or anticipated procedural difficulty.

This study used an outcome assessor-blind assessment design. The 2 senior anesthesiologists who assessed clinical performance from the deidentified videos were blinded to the trainee group allocation. Patients were not informed about the trainees’ training modality. Supervising anesthesiologists were not involved in outcome scoring and intervened only when necessary for patient safety. Because the VR platform and manikin training were visually and operationally distinct, trainees and instructors could not be blinded to the training allocation; however, neither trainees nor instructors participated in video-based outcome scoring. To ensure fairness after outcome assessment, trainees were offered the opportunity to receive training with an alternative modality after completing all study procedures and evaluations.

### Interventions

#### General Training Procedures

The interventions were described with reference to the Template for Intervention Description and Replication (TIDieR) framework to improve replicability. The participants were assigned to either a VR-based training group (SG) or a traditional manikin-based training group (CG). Both groups received structured training designed to teach CSE anesthesia techniques. Training in both groups was delivered face-to-face in a dedicated simulation laboratory by experienced anesthesiology instructors using standardized teaching scripts and feedback principles. Each participant completed a standardized 7-day intensive training curriculum, consisting of five 45-minute training sessions per day, with a 15-minute interval between consecutive sessions. No modifications were made to the intervention protocols during the trial, and all randomized participants completed their assigned training sessions. No additional simulation-based training related to CSE anesthesia was provided during the study period. Patients received routine perioperative care according to institutional practices. Use of the assigned intervention was operationalized as completion of the standardized 7-day curriculum, consisting of 35 supervised training sessions. Because training was conducted only during scheduled face-to-face laboratory sessions, intervention use was monitored by session completion rather than by web logins, logfile analysis, or automated session analytics.

#### SG

Trainees used the Haptic-VR CSE Teaching Platform. The system hardware included an Oculus Rift S headset (Meta Platforms, Inc) for 3D visualization and a customized Geomagic Touch haptic device (3D Systems, Inc) to render force feedback. The platform reproduced the layered resistance of anatomical structures encountered during neuraxial procedures, including the supraspinous ligament, interspinous ligament, and ligamentum flavum, as well as the characteristic LOR sensation. The purpose of this intervention was to allow trainees to repeatedly practice needle advancement, trajectory control, tissue-layer discrimination, and tactile recognition of LOR in a controlled and reproducible environment. For this trial, the hardware and software configuration, training tasks, and instructional content were set before participant enrollment. No functional changes, content updates, major bug fixes, system failures, or downtime occurred during the trial.

Training tasks included repeated virtual punctures under two modes: (1) a visualization-assisted mode with transparent anatomical overlays to facilitate understanding of 3D spatial relationships, and (2) a blinded mode without visual guidance to enhance reliance on tactile feedback. Aseptic procedural steps (eg, disinfection and draping) were represented through interface-based interactions rather than physical manipulation. In contrast, the manikin group allowed physical rehearsal of the aseptic workflow. This design allowed the VR curriculum to focus primarily on haptic skill acquisition and spatial-technical performance. During each session, experienced anesthesiology instructors provided standardized guidance and feedback. The platform displayed procedural information, including puncture depth and angular deviation, during puncture training, but it did not provide automated clinical decision support, adaptive training recommendations, or algorithm-generated feedback. Therefore, instructor guidance and feedback were part of the trial intervention rather than an automated platform function.

#### CG

Trainees practiced using a standard lumbar puncture manikin (Tuoren Medical). The manikin-based curriculum emphasized patient positioning, surface landmark palpation, identification of the puncture interspace, aseptic preparation, draping, procedural workflows, and needle manipulation. In contrast to the VR curriculum, manikin-based training allowed for physical rehearsal of sterile preparation and procedural sequencing. During each session, the same instructor team provided standardized feedback on positioning, sterile workflow, puncture mechanics, and needle handling. The training dose was identical to that used in the SG.

### Clinical Assessment and Procedure Evaluation

After completing the training curriculum, each trainee performed 1 supervised CSE anesthesia procedure on an eligible patient according to the prespecified clinical assessment sequence defined in the randomization procedure. The procedure was conducted under the direct supervision of an attending anesthesiologist, who intervened only when necessary for patient safety. The entire procedure was video-recorded. Videos were edited to remove audio or visual information that could reveal the trainees’ training allocation. The camera angle was standardized to focus on the lumbar puncture field and the operator’s hands, thereby minimizing the possibility of identifying trainees.

Clinical performance was assessed using the Modified Spinal-Epidural Puncture Assessment Scale (Multimedia Appendix 1). The core domains and scoring structure of this scale were adapted from established procedural assessment frameworks, including the Global Rating Scale (GRS), Direct Observation of Procedural Skills (DOPS), and prior anesthesia procedural assessment approaches that combine task-specific checklists with GRS [27-29]. Based on these frameworks and in accordance with standardized residency training requirements and institutional procedural teaching standards for neuraxial anesthesia, modifications were made to reflect the key procedural steps of CSE anesthesia. This approach is also consistent with recent neuraxial training literature emphasizing the structured assessment of lumbar puncture, epidural anesthesia, and spinal anesthesia skills [24]. These domains included patient positioning, disinfection scope and sequence, LOR recognition using the bubble compression test, ligamentum flavum penetration, avoidance of arachnoid membrane puncture, and aseptic technique compliance.

The scoring domains and point values were predefined before the outcome assessment and were based on the clinical importance of each procedural step for procedural success and patient safety. Greater weight was assigned to critical CSE-specific technical steps, including LOR recognition, ligamentum flavum penetration, avoidance of arachnoid membrane puncture, and aseptic technique compliance. The total score ranged from 0 to 100, with higher scores indicating better procedural performance. To improve scoring transparency, operational scoring anchors for full, partial, and 0-score performance are provided in Multimedia Appendix 1. Although each item had a predefined maximum score, raters were allowed to assign partial scores, including half-point increments, when performance partially met the predefined criteria or reflected intermediate performance quality.

Two senior anesthesiologists, who were not involved in the training intervention, independently assessed all study videos and were blinded to group allocation. Before the formal assessment, the raters reviewed the scoring criteria together to standardize the interpretation of each domain. The final score for each performance domain was calculated as the mean of the 2 raters’ scores.

### Outcomes

#### Primary Outcome

Total clinical performance score on the Modified Spinal-Epidural Puncture Assessment Scale, ranging from 0 to 100.

#### Secondary Outcomes

Number of active/passive angle adjustments, time from puncture initiation to breakthrough of the ligamentum flavum, first-attempt success rate, and complications within 24 hours after the procedure. Complications assessed within 24 hours included postdural puncture headache, local bleeding, infection, nerve injury, and intracranial hypotension syndrome. All outcomes were obtained through video-based expert assessments, procedural records, and postoperative clinical follow-ups; no online questionnaires or participant self-assessments were used as outcome measures.

Harms were assessed during the supervised CSE procedure and within 24 hours postoperatively by the supervising anesthesiologist and study team. The prespecified harms included accidental dural puncture, postdural puncture headache, local bleeding or hematoma, infection, nerve injury, and intracranial hypotension syndrome. Intervention-related adverse events associated with the training procedures were also recorded. For the VR intervention, training-related adverse events included VR-related discomfort, privacy breaches, data security incidents, system failure, downtime, and other technical problems during platform use.

### Sample Size

As this was a pilot feasibility study, the sample size was determined based on practical resource constraints and sample sizes used in recent pilot studies of simulation-based medical education [30,31]. Consistent with these pilot studies, a sample size of 10 participants per group was selected. This sample size was considered appropriate for a pilot study to estimate the variability of the primary outcome and generate preliminary effect estimates that could inform outcome selection and sample size planning for a subsequent adequately powered trial. No additional attrition allowance was applied to the pilot sample size because the sample size was determined by feasibility considerations, and all training and assessment sessions were scheduled and supervised. No interim analyses or stopping guidelines were planned or conducted because of the pilot nature and short duration of the study.

### Statistical Methods

Statistical analyses were performed using SPSS software (version 25.0; IBM Corp). All randomized participants were included in the primary analysis according to the intention-to-treat principle and were analyzed in the groups to which they were originally assigned. Because all randomized participants completed the assigned training intervention and clinical outcome assessment, the intention-to-treat and per-protocol populations were identical.

Missing data were assessed at both the participant and item levels. Participant-level missing data were defined as attrition, withdrawal, loss to follow-up, or failure to complete clinical assessments. Item-level missing data were defined as incomplete or unavailable values for individual outcome measures or assessment items. Because no participant-level or item-level missing data occurred, the missing-data proportion was 0% at both levels. Therefore, a missing completely at random test, multiple imputation, or other missing-data handling procedures were not performed.

The interrater reliability for the total clinical performance score between the 2 blinded assessors was evaluated using a 2-way random-effects, absolute-agreement intraclass correlation coefficient (ICC). Because the final performance scores were based on the mean of the 2 raters’ scores, the average-measures ICC was used to indicate the reliability of the final analyzed scores.

Baseline comparability between the 2 groups was assessed using absolute standardized mean differences (SMDs), where an SMD<0.25 was considered to indicate an adequate balance of covariates. In accordance with the CONSORT statement for randomized trials, significance testing (*P* values) was not performed for baseline characteristics, as any observed differences were due to chance following randomization. The Shapiro-Wilk test was used to assess the normality of continuous data. Normally distributed data were expressed as means (SD) and analyzed using Student’s 2-tailed *t* test. Nonnormally distributed or ceiling-effect outcomes were summarized as medians with IQRs. Quartiles were calculated using Tukey hinges. Between-group comparisons for these outcomes were performed using the Mann-Whitney *U* test, with exact 2-sided *P* values reported because of the small sample size. Categorical data were presented as n (%), and comparisons were performed using Fisher exact test. Effect estimates were reported with corresponding 95% CIs where appropriate. For normally distributed continuous outcomes, between-group differences were expressed as mean differences with 95% CIs. For nonnormally distributed or ceiling-effect outcomes, between-group differences were expressed as Hodges-Lehmann median differences with 95% CIs. For categorical outcomes, between-group differences were expressed as risk differences with 95% CIs where applicable. Cohen *d* was calculated for normally distributed continuous outcomes to inform interpretation and future sample size estimation but was not calculated for nonnormally distributed or ceiling-effect outcomes.

No formal adjustment for multiple comparisons was performed. Given the pilot and exploratory nature of this study, analyses of secondary outcomes were considered hypothesis-generating, and these findings should be interpreted cautiously. No subgroup, sensitivity, or ancillary analyses were prespecified or performed. A 2-sided *P* value of<.05 was considered statistically significant.

## Results

### Participant Flow

A total of 20 anesthesiology interns were assessed for eligibility and randomized in a 1:1 ratio to the CG (n=10) or the SG (n=10). All 20 randomized participants completed the assigned training intervention and subsequent clinical assessment. This corresponded to the completion of the full assigned training dose in both groups, defined as 35 supervised training sessions per trainee. No participants withdrew, were lost to follow-up, or were excluded from the analysis. No participant-level missing data or item-level missing data were observed; therefore, the missing data proportion was 0% at both levels (Figure 1).

**Figure 1.**
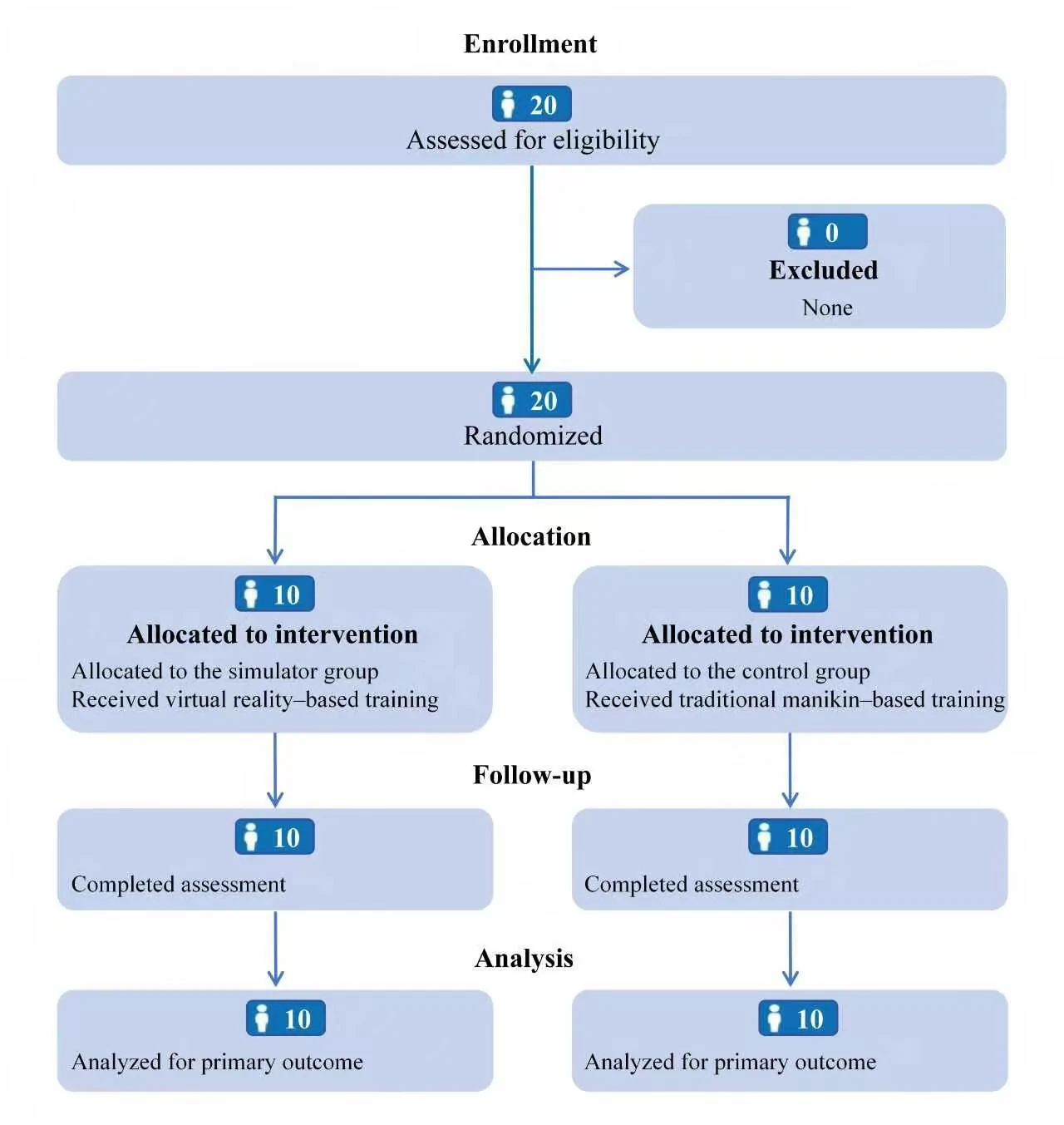
CONSORT (Consolidated Standards of Reporting Trials) participant flow diagram of anesthesiology trainees in a single-center, prospective, parallel-group randomized pilot trial comparing haptic virtual reality–based training vs traditional manikin-based training for combined spinal-epidural anesthesia at Fujian Provincial Hospital, Fuzhou, China, March 2021.

### Baseline Data

The baseline demographic characteristics and prior clinical experience are presented in Table 1. Socioeconomic status and computer, internet, or eHealth literacy were not collected as baseline variables because the intervention was delivered in supervised, face-to-face laboratory sessions rather than through remote, self-directed access. The 2 groups were comparable in terms of all measured variables. The absolute SMDs for the baseline characteristics are provided in Multimedia Appendix 2.

**Table 1.** Baseline characteristics of anesthesiology trainees enrolled in a single-center, prospective, parallel-group randomized pilot trial comparing haptic virtual reality–based training vs traditional manikin-based training for CSE^a^ anesthesia at Fujian Provincial Hospital, Fuzhou, China, March 2021.

Characteristic	CG^b^ (n=10)	SG^c^ (n=10)	SMD^d^
Age (y), mean (SD)	22.50 (0.53)	22.60 (0.52)	0.19
Sex			0.20
Male	6	5	
Female	4	5	
Handedness			0.33
Right	9	10	
Left	1	0	
Prior CSE procedures, mean (SD)	1.2 (0.8)	1.4 (0.9)	0.24
Prior lumbar punctures, mean (SD)	3.5 (1.2)	3.2 (1.4)	0.23

a CSE: combined spinal-epidural.

b CG: control group.

c SG: simulator group.

d SMD: standardized mean difference.

### Primary Outcome: Technical Skill Acquisition

Interrater reliability for the primary outcome, the total clinical performance score, was excellent, with an average-measures ICC of 0.977. The SG had a higher total clinical performance score than the CG, with a mean difference of 4.50 points (95% CI 1.85-7.15; *P*=.002; Cohen *d*=1.59; Table 2). Exploratory domain-specific analyses showed higher SG scores in haptic-dependent components, including the bubble compression test (mean difference 1.90 points, 95% CI 1.14- 2.66; *P*<.001; Cohen *d*=2.37) and ligamentum flavum penetration (mean difference 1.90 points, 95% CI 1.18 to 2.62; *P*<.001; Cohen *d*=2.48). In contrast, the SG had lower aseptic technique compliance scores than the CG (mean difference −1.40 points, 95% CI −2.00 to −0.80; *P*<.001; Cohen *d*=−2.18), suggesting a domain-specific pattern of training effects.

**Table 2.** Clinical performance scores of anesthesiology trainees during supervised combined spinal-epidural anesthesia procedures on real patients, by training modality^a^.

Performance domain	CG^b^	SG^c^	Effect estimate, MD^d^ (95% CI)	*P* value	Cohen *d*
Patient positioning (8 points), mean (SD)	5.00 (0.67)	6.40 (1.07)	1.40 (0.56 to 2.24)	.003	1.56
Scope and sequence of disinfection (5 points), mean (SD)	3.20 (0.42)	3.70 (0.82)	0.50 (–0.11 to 1.11)	.12	0.77
Bubble compression (LOR^e^; 10 points), mean (SD)	5.80 (0.92)	7.70 (0.67)	1.90 (1.14 to 2.66)	<.001	2.37
Ligamentum flavum penetration (10 points), mean (SD)	5.90 (0.74)	7.80 (0.79)	1.90 (1.18 to 2.62)	<.001	2.48
Epidural needle not penetrating the arachnoid membrane (10 points), median (IQR)	10.00 (10.00‐10.00)	10.00 (10.00‐10.00)	Hodges-Lehmann median difference 0.00 points (0.00-0.00)	.74	—^f^
Compliance with aseptic technique (10 points), mean (SD)	7.90 (0.57)	6.50 (0.71)	–1.40 (–2.00 to –0.80)	<.001	–2.18
Total score (100 points), mean (SD)	86.70 (2.87)	91.20 (2.78)	4.50 (1.85 to 7.15)	.002	1.59

a Data are from a single-center, prospective, parallel-group randomized pilot trial comparing haptic virtual reality–based training vs traditional manikin-based training for anesthesiology trainees at Fujian Provincial Hospital, Fuzhou, China, March 2021. Trainees were assessed during supervised procedures on real patients. For each domain, the final score was calculated as the mean of the 2 independent blinded raters' scores. Effect estimates were calculated as SG minus CG and are reported as mean differences or Hodges-Lehmann median differences, as appropriate. Exact 2-sided *P* values were reported for Mann-Whitney *U* tests. Cohen *d* was not calculated for the ceiling-effect outcome.

b CG: control group.

c SG: simulator group.

d MD: Mean difference.

e LOR: loss of resistance.

f Not applicable.

### Secondary Outcomes

#### Efficiency and Safety Metrics

The procedural performance outcomes are shown in Table 3. The time from puncture initiation to breakthrough of the ligamentum flavum was shorter in the SG than in the CG, with a mean difference of −2.75 (95% CI −3.89 to −1.61; *P*<.001) minutes. For puncture-angle adjustments, the SG required more active adjustments than the CG (Hodges-Lehmann median difference 1.0 adjustment, 95% CI 0.0-2.0; *P*=.03), whereas passive adjustments due to bone contact were fewer in the SG (Hodges-Lehmann median difference −2.0 adjustments, 95% CI −2.0 to −1.0; *P*<.001). The first attempt success was numerically higher in the SG than in the CG (6/10, 60% vs 3/10, 30%); however, the CI was wide and crossed the null value (risk difference 30 percentage points, 95% CI −11.8 to 60.1; *P*=.37), indicating substantial imprecision. Given the pilot sample size and exploratory nature of these secondary outcomes, these procedural findings should be interpreted cautiously.

**Table 3.** Procedural performance outcomes of anesthesiology trainees during supervised combined spinal-epidural anesthesia procedures on real patients, by training modality^a^.

Item	CG^b^	SG^c^	Effect estimate (95% CI)	*P* value
First attempt success, n (%)	3 (30)	6 (60)	Risk difference, 30 percentage points (95% CI −11.8 to 60.1)	.37
Number of active puncture-angle adjustments, median (IQR)	1.0 (1.0‐2.0)	2.0 (2.0‐3.0)	Hodges-Lehmann median difference 1.0 adjustment, (95% CI 0.0 to 2.0)	.03
Number of passive puncture-angle adjustments, median (IQR)	2.5 (2.0‐3.0)	1.0 (0.0‐1.0)	Hodges-Lehmann median difference –2.0 adjustments, (95% CI −2.0 to −1.0)	<.001
Time from puncture initiation to breakthrough of ligamentum flavum, mean (SD), min	8.66 (1.19)	5.91 (1.24)	Mean difference −2.75, min (95% CI −3.89 to −1.61)	<.001

a Data are from a single-center, prospective, parallel-group randomized pilot trial comparing haptic virtual reality–based training vs traditional manikin-based training for anesthesiology trainees at Fujian Provincial Hospital, Fuzhou, China, March 2021. Trainees were assessed during supervised procedures on real patients. Effect estimates were calculated as SG minus control group and are reported as risk differences, mean differences, or Hodges-Lehmann median differences, as appropriate. Exact 2-sided *P* values were reported for Mann-Whitney *U* tests.

b CG: control group.

c SG: simulator group.

#### Harms

No major complications, including accidental dural puncture, nerve injury, local bleeding or hematoma, infection, postdural puncture headache, or intracranial hypotension syndrome, were observed in either group during the supervised procedure or within the 24-hour postoperative follow-up period. No intervention-related adverse events were reported (0/10 and 0/10 in the control and SGs, respectively). No participants withdrew because of any harm. No VR-related discomfort, privacy breach, data-security incident, system failure, downtime, or other technical problem affecting training delivery was observed or reported during platform use.

## Discussion

### Key Findings

This randomized controlled pilot study aimed to investigate whether haptic-VR training can facilitate the early transfer of CSE anesthesia procedural skills to supervised procedures on real patients and whether the effects of this training differ across various procedural dimensions.

Compared with training using manikins, trainees who received haptic-VR training demonstrated better overall procedural performance, as assessed by the study’s specific scale, during their first supervised clinical CSE procedure. Exploratory dimensional analysis suggested that the haptic-VR training group performed better at recognizing the LOR and ligamentum flavum penetration, while the manikin training group performed better in aseptic technique. Indicators related to puncture efficiency and needle trajectory adjustment were consistent in direction with the improved needle-path control observed after haptic-VR training; however, the precision of the effect estimates for first-attempt success rates was insufficient to draw definitive conclusions. Overall, these results provide stronger support for the notion that “different training methods have specific advantages across different skill dimensions” rather than indicating that any single simulation training method possesses a universal advantage.

### Interpretation of Results and Comparison With Previous Studies

Most published studies on VR and XR in the fields of anesthesiology and intraspinal anesthesia education primarily evaluate learners’ performance in simulated environments or rely on self-reported outcomes; therefore, it remains unclear whether these skills can be effectively transferred to clinical practice [17-24]. A recent systematic review on intraspinal anesthesia training also noted significant heterogeneity among studies in terms of teaching methods and assessment approaches, and high-quality evidence directly linking simulation training to performance in real patients remains limited [24].

Previous evaluations of the platform used in this study have shown that trainees are able to follow a certain learning curve with virtual patients, demonstrate a positive experience with repeated practice, as well as mastery of the puncture procedure steps; however, those studies did not compare different training modalities or further evaluate trainees’ performance with real patients [32]. By using a randomized controlled design and having blinded evaluators score videos of the first supervised clinical CSE procedure, this study brought the outcome measures closer to performance in real clinical settings. However, a single, immediate, supervised clinical procedure represents only “near transfer” under relatively controlled conditions [33], and does not yet demonstrate that trainees have acquired lasting procedural competence, are capable of performing the procedure independently, or can improve patient outcomes.

The high overall clinical performance scores observed in this study should be interpreted with caution in light of the above. A recent meta-analysis incorporating randomized studies found no significant overall difference in procedural skill outcomes between virtual simulation and training using manikins or live participants, and noted substantial heterogeneity among different interventions and assessment methods [34]. Therefore, the results of this study do not demonstrate that VR is superior to traditional simulation training overall. Rather, these results suggest that training outcomes may depend on whether the features of a particular simulator align with the specific skill components ultimately being assessed [34,35]. It is particularly important to note that the total scores in this study were derived from study-specific scales, and it has not yet been determined which score differences can be considered pedagogically or clinically meaningful.

The exploratory advantages observed in the recognition of the LOR and puncture of the ligamentum flavum are reasonable, as VR courses can repeatedly provide trainees with standardized force feedback cues while allowing them to control the needle trajectory within a 3D model [32]. In contrast, repeated puncture of physical models may alter the resistance characteristics of the material itself and create preexisting needle tracks, whereas digital force-feedback models can provide more consistent resistance feedback across multiple practice sessions [16,32]. However, the VR intervention in this study did not consist solely of haptic feedback; it also incorporated transparent anatomical displays, depth and angle information, and instructor guidance. Therefore, it is currently impossible to determine whether the observed advantages stemmed from haptic feedback, visual-spatial guidance, repeated practice, or a combination of these factors.

The results regarding procedure duration and needle angle adjustments are consistent with more efficient control of the needle trajectory, but this has not yet been proven. Fewer passive adjustments following bone contact may indicate that trainees were better able to anticipate the needle path; conversely, more active adjustments may reflect either conscious corrections by the trainees or greater uncertainty during the procedure. Since these process-based indicators in this study have not yet been validated as independent measures of procedural proficiency, nor have they been shown to correlate with patient outcomes, they should still be considered exploratory findings. Future research could evaluate these indicators in conjunction with objective trajectory parameters, the number of errors, the frequency of instructor interventions, and predefined criteria for successful needle insertion control.

The finding that the VR training group performed poorly in aseptic technique is also significant. In the VR environment, aseptic preparation and draping are primarily performed through interface operations, whereas the manikin training group physically practiced these steps. This illustrates a classic “training specificity effect.” The closer a training method is to a real-world task, the more likely it is to facilitate the learning of that task [36]. A recent randomized study on central venous catheterization training also supports the complementary roles of VR and manikin training, although the areas of advantage across different skill dimensions differed from those in this study: the VR component of that study placed greater emphasis on standardized operating procedures, whereas manikin training was more beneficial for haptic-related operations and postoperative tasks [35]. These differences may stem from variations in the simulator design, haptic feedback capabilities, curriculum content, and assessment methods [34,35]. Taken together, these studies suggest that VR and manikin training should not be regarded as entirely identical or interchangeable interventions; rather, the “functional realism” of different simulation methods across specific skill components should be evaluated further [34-36].

The accuracy of the estimates of the first-attempt success rates was insufficient; therefore, it is not yet possible to determine whether either training method truly improved procedural success rates. Similarly, the absence of adverse events during the short-term follow-up period cannot be interpreted as evidence that the 2 training methods have comparable safety profiles. All procedures in this study were performed under supervision, and the patients were screened. Furthermore, the sample size of this trial was insufficient to detect low-incidence complications. Future confirmatory studies should predefine outcomes, such as clinical procedure success, instructor rescue interventions, patient-reported experiences, and procedure-related adverse events, and establish appropriate follow-up time frames based on the characteristics of the complications of interest.

### Educational Implications and Implementation Insights

The primary educational implication of this study is not “replacement” but rather “sequential application.” Haptic-VR may be more suitable for repetitive practice of specific procedural skills, such as identifying tissue resistance, spatial orientation, and needle-path control; this can be followed by training with physical manikins to integrate patient positioning, instrument manipulation, aseptic preparation, catheter insertion steps, and the complete procedural workflow, after which trainees can progress to the supervised patient procedure phase [34-36]. However, this sequential training model remains a hypothesis based on existing findings and should be validated through future research; it cannot be directly regarded as a proven best training approach. Future comparisons between hybrid “VR combined with manikins” training programs and single-manikin programs of equivalent duration will provide a more direct means of assessing whether this complementary training model offers added value.

Course design should also be based on the level of procedural competence achieved rather than solely on the duration or number of training sessions [37]. In the future, clear proficiency standards could be established; for example, trainees could first be required to demonstrate consistent haptic discrimination and needle trajectory control in a VR environment, followed by satisfactory performance of the complete procedure in a validated physical manikin assessment. Prior to routine implementation, training programs require further evaluation of instructor time commitments, hardware and maintenance costs, system availability, motion sickness or VR-related discomfort, and accessibility [38], as well as training performance under varying anatomical conditions. This study did not evaluate the aforementioned implementation outcomes. Furthermore, because the intervention used a centralized training model under close instructor supervision, its feasibility in routine training environments or unsupervised self-directed training cannot be directly inferred.

Future multicenter studies should predefine clinically meaningful minimum differences [39] and use validated outcome assessment tools [40]; they should also include a more diverse cohort of trainees and patients and control for differences in case difficulty through stratification, balancing, or statistical adjustments. Furthermore, trainees’ performance in completing multiple procedures on real patients should be assessed continuously over a defined time period rather than focusing solely on the first procedure. The study design should also clearly distinguish between primary and exploratory outcomes [25], appropriately control for multiple comparisons, and simultaneously evaluate skill retention and immediate performance [41]. Only such a study design can further determine whether the dimension-specific effects observed in this study are reproducible and whether these advantages ultimately translate into sustained clinical competence or patient benefits.

### Limitations

This study had several limitations. First, as a small-sample, single-center pilot trial, the precision of the effect estimates for secondary and categorical outcomes was limited [42]. Although randomization can reduce allocation bias, differences in the baseline characteristics between the 2 groups of trainees may exist, given the small sample size. There is no guarantee that the baseline characteristics were evenly distributed across groups [43]. Furthermore, since each trainee was evaluated on different patients, variations in patient anatomy [44] and the complexity of the procedures may have influenced the trainees’ performance. This study was not designed to assess rare adverse events.

Second, each trainee completed only 1 clinical assessment shortly after the training concluded; neither pretraining clinical performance data served as a baseline, nor was a follow-up assessment of skill retention conducted. Therefore, the study results primarily reflect the trainees’ performance during their first supervised clinical procedure following training and cannot be used to evaluate the extent of skill improvement, consistency across cases, independent competence [33], or long-term retention of skills [41]. Furthermore, the patient population included in this study limits the generalizability of the findings to populations characterized by advanced age, high BMI, challenging anatomy, and other complex clinical cases.

Third, the modified scale was derived from an established assessment framework. Agreement between raters was high, but the modified version has not yet undergone formal psychometric validation. The present study also cannot determine what size of score difference would be clinically or educationally meaningful. Some domain-specific analyses and other secondary outcomes were exploratory, with no correction for multiple comparisons. Therefore, the differences seen in these analyses provide signals for hypothesis generation rather than confirmatory evidence [45].

### Conclusions and Educational Implications

In this pilot trial, trainees who used haptic-feedback VR showed an early signal of transfer for selected tactile-dependent CSE skills during supervised patient procedures. Because the outcome was clinical performance rather than simulator scoring alone, the data are closer to early patient-care transfer than much of the existing simulation literature. This pattern was not a simple advantage for VR. Haptic-feedback VR appeared better aligned with tactile judgment and needle-path control, whereas manikin-based training remained important for workflow and aseptic practices. A practical curriculum would use the 2 tools for different purposes: VR for repeated exposure to resistance cues and physical simulation for the manual sequence and sterile steps of CSE. A larger multicenter trial would be a more appropriate test of this model, with repeated patient assessments, longer follow-up, validated scoring tools, and a prespecified approach to multiple testing.

## Supplementary material

10.2196/92207 Multimedia Appendix 1 Modified Spinal-Epidural Puncture Assessment Scale.

10.2196/92207 Multimedia Appendix 2 Patient baseline characteristics.

10.2196/92207 Multimedia Appendix 3 Haptic virtual reality training platform and interface for combined spinal-epidural anesthesia training.

10.2196/92207 Checklist 1 CONSORT 2025 expanded checklist.

10.2196/92207 Checklist 2 CONSORT-EHEALTH checklist (V 1.6.1).

## Abbreviations

CG: control group
CONSORT: Consolidated Standards of Reporting Trials
CONSORT-AI: Consolidated Standards of Reporting Trials–AI
CONSORT-EHEALTH: Consolidated Standards of Reporting Trials of Electronic and Mobile Health Applications and Online Telehealth
CSE: combined spinal-epidural
DOPS: Direct Observation of Procedural Skills
GRS: Global Rating Scale
ICC: intraclass correlation coefficient
LOR: loss of resistance
SG: simulator group
SMD: standardized mean differences
TIDieR: Template for Intervention Description and Replication
VR: virtual reality
XR: extended reality
