# Supplementary material for: Haptic Virtual Reality Training for Combined Spinal-Epidural Anesthesia in Anesthesiology Trainees: Prospective Randomized Controlled Pilot Trial
Source: JMIR Serious Games. 2026 Oct 8;14:e92207. doi: 10.2196/92207 (PMC13649171; doi:10.2196/92207)
Supplement: Supplementary file 2 [file games-v14-e92207-s005.pdf]

CONSORT-EHEALTH checklist (V.1.6.1): Information to include when reporting eHealth/mHealth trials (web‐based/Internet‐based intervention and decision aids, but also social media, serious games, DVDs, mobile applications, certain telehealth applications)

Section/Topic	Item No.	CONSORT* Checklist Item	EHEALTH Extensions (additions to, or clarification of the CONSORT item)	Importance	Reported on page No
**TITLE & ABSTRACT**					
Title & Abstract	1a	Identification as a randomized trial in the title	i) Identify the mode of delivery in the title. Preferably use "web-based" and/or "mobile" and/or "electronic game" in the title. Avoid ambiguous terms like "online", "virtual", "interactive". Use "Internet-based" only if the intervention includes non-web-based Internet components (e.g., email); use "computer-based" or "electronic" only if offline products are used. Use "virtual" only in the context of "virtual reality" (3-D worlds). Use "online" only in the context of "online support groups". Complement or substitute product names with broader terms for the class of products, especially if the application runs on different platforms.	*Essential*	**p 1**
			ii) Mention non-web-based components or important co-interventions in the title, if any (e.g., "with telephone support").	*Highly Recommended*	**No additional title-level co-intervention; offline face-to-face delivery is reported on pp 2, 7, and 11.**
			iii) Mention primary condition or target group in the title, if any (e.g., "for children with Type I Diabetes"). Example: A Web-based and Mobile Intervention with Telephone Support for Children with Type I Diabetes: Randomized Controlled Trial.	*Essential*	**p 1**
	1b	Structured summary of trial design, methods, results, and conclusions NPT** extension: Description of experimental treatment, comparator, care providers, centers, and blinding status	Methods in abstract: i) Mention key features/functionalities/components of the intervention and comparator in the abstract. If possible, also mention theories and principles used for designing the site. Keep in mind the needs of systematic reviewers and indexers by including important synonyms. Only report in the abstract what the main paper is reporting.	*Essential*	**p 2**
			ii) Clarify the level of human involvement in the abstract, e.g., use phrases like "fully automated" vs. "therapist/nurse/care provider/physician-assisted". Mention number and expertise of providers involved, if any. Only report in the abstract what the main paper is reporting.	*Highly Recommended*	**p 2**
			iii) Open vs. closed, web-based self-assessment vs. face-to-face assessments in abstract: Mention how participants were recruited (online vs. offline), e.g., from an open access website, from a clinic, or from a closed online user group, and clarify whether this was a purely web-based trial or whether there were face-to-face components. Clearly say if outcomes were self-assessed through questionnaires. To avoid confusion, use "blinded" or "unblinded" to indicate the level of blinding instead of "open".	*Highly Recommended*	**p 2; offline recruitment and face-to-face delivery/assessment are further described on p 7.**
			iv) Results in abstract must contain use data: Report number of participants enrolled/assessed in each group and the use/uptake of the intervention (e.g., attrition/adherence metrics, use over time, number of logins), in addition to primary/secondary outcomes.	*Highly Recommended*	**pp 2–3**
			v) Conclusions/discussions in abstract for negative trials: Discuss the primary outcome. If the trial is negative and the intervention was not used, discuss whether negative results are attributable to lack of uptake and discuss reasons.	*Highly Recommended*	**Not applicable; this was not a negative trial. Primary outcome and conclusions are reported on pp 2–3.**
**INTRODUCTION**					
Background and objectives	2a	Scientific background and explanation of rationale	i) Describe the problem and the type of system/solution that is the object of the study: intended as a stand-alone intervention vs. incorporated in a broader health care program; intended for a particular patient population; goals of the intervention, e.g., being more cost-effective than other interventions, replacing or complementing other solutions. Details about the intervention are provided in Methods under item 5.	*Essential*	**pp 4–6**
			ii) Scientific background and rationale: Describe what is known about the type of system that is the object of the study, including use of similar systems for other conditions/diagnoses if appropriate; motivation for the study; reasons and context for this specific study; stakeholder viewpoint; potential impact of findings. Briefly justify the choice of comparator.	*Essential*	**pp 4–6**

Section/Topic	Item No.	CONSORT* Checklist Item	EHEALTH Extensions (additions to, or clarification of the CONSORT item)	Importance	Reported on page No
	2b	Specific objectives or hypotheses	No EHEALTH-specific additions here. Note: contrary to STARE-HI, CONSORT-EHEALTH does not recommend mentioning IRB approval in this section; JMIR and other journals typically recommend this as a subheading under Methods. CONSORT-EHEALTH has a separate item for ethical considerations.		**p 6**
**METHODS**					
Trial design	3a	Description of trial design (such as parallel, factorial) including allocation ratio	No EHEALTH-specific additions here.		**pp 6–7**
	3b	Important changes to methods after trial commencement (such as eligibility criteria), with reasons	i) Bug fixes, downtimes, content changes: eHealth systems are often dynamic systems. A description of changes to methods therefore also includes important changes made to the intervention or comparator during the trial, e.g., major bug fixes or changes in functionality or content, and other unexpected events that may have influenced study design, such as staff changes, system failures/downtimes, etc.	*Highly Recommended*	**pp 7, 11–12, and 22**
Participants	4a	Eligibility criteria for participants	i) Computer/Internet literacy is often an implicit de facto eligibility criterion and should be explicitly clarified.	*Highly Recommended*	**pp 8–9**
			ii) Open vs. closed, web-based vs. face-to-face assessments: Mention how participants were recruited (online vs. offline), e.g., from an open access website or from a clinic, and clarify whether this was a purely web-based trial or whether there were face-to-face components. In online-only trials, clarify if participants were quasi-anonymous and whether multiple identities were possible or whether technical/logistical measures such as cookies, email confirmation, or phone calls were used to detect or prevent these.	*Essential*	**pp 7–9**
			iii) Information given during recruitment: Specify how participants were briefed for recruitment and informed consent procedures, e.g., publish the informed consent documentation as an appendix, because this information may affect user self-selection, user expectation, and may bias results.	*Highly Recommended*	**pp 7–9**
	4b	Settings and locations where the data were collected	i) Clearly report whether outcomes were self-assessed through online questionnaires, as common in web-based trials, or otherwise.	*Essential*	**pp 6–7 and 13–16**
			ii) Report how institutional affiliations are displayed to potential participants on eHealth media, as affiliations with prestigious hospitals or universities may affect volunteer rates, use, and reactions to an intervention. This is not a required item; describe only if this may bias results.	*Recommended*	**Not applicable; see p 7**
Interventions	5	The interventions for each group with sufficient details to allow replication, including how and when they were actually administered	i) Mention names, credentials, affiliations of the developers, sponsors, and owners. If authors/evaluators are owners or developers of the software, this needs to be declared in a Conflict of Interest section or mentioned elsewhere in the manuscript.	*Highly Recommended*	**pp 11–13 and 30–32**
			ii) Describe the history/development process of the application and previous formative evaluations, e.g., focus groups or usability testing, because these affect adoption/use rates and help with interpreting results.	*Highly Recommended*	**Previous development and application of the platform are cited, and the study team's relationship to the platform is disclosed on pp 30–31 and in reference 32 on p 37; no additional formative usability evaluation was conducted for this trial.**
			iii) Revisions and updating: Clearly mention the date and/or version number of the application/intervention and comparator, if applicable, evaluated; describe whether the intervention underwent major changes during the evaluation process or whether development/content was frozen during the trial. Describe dynamic components such as news feeds or changing content that may affect replicability.	*Highly Recommended*	**pp 11–12**

Section/Topic	Item No.	CONSORT* Checklist Item	EHEALTH Extensions (additions to, or clarification of the CONSORT item)	Importance	Reported on page No
			iv) Provide information on quality assurance methods to ensure accuracy and quality of information provided, if applicable.	*Highly Recommended*	**The platform configuration, training tasks, and instructional content were fixed before enrollment, and no functionality changes, major bug fixes, system failures, or downtime occurred during the trial; see pp 12 and 22.**
			v) Ensure replicability by publishing the source code and/or providing screenshots/screen-capture video and/or flowcharts of algorithms used. Replicability is a hallmark of scientific reporting.	*Highly Recommended*	**pp 30 and 39**
			vi) Digital preservation: Provide the URL of the application, and because the intervention may change or disappear over the years, ensure that the intervention is archived using Internet Archive, webcitation.org, and/or by publishing source code or screenshots/videos alongside the article. As pages behind login screens cannot be archived, consider creating demo pages accessible without login.	*Highly Recommended*	**pp 30 and 39**
			vii) Access: Describe how participants accessed the application, in what setting/context, whether they had to pay or were paid, and whether they had to be members of a specific group. If known, describe how participants obtained access to the platform and Internet. To ensure access for editors/reviewers/readers, consider providing a backdoor login account or demo mode.	*Essential*	**pp 7–8, 11–12, and 30**
			viii) Describe mode of delivery, features/functionalities/components of the intervention and comparator, and the theoretical framework used to design them, including instructional strategy, behaviour change techniques, persuasive features, content origin and development, tailoring, user progress tracking and feedback, communication delivery channels, synchronous/asynchronous communication, and presentation strategies such as page design principles, average text per page, and hyperlinks.	*Essential*	**pp 11–13 and 39**
			ix) Describe use parameters, e.g., intended doses and optimal timing for use. Clarify instructions or recommendations given to users regarding timing, frequency, or intensity of use, if any, or whether the intervention was used ad libitum.	*Highly Recommended*	**pp 11–12 and 20**
			x) Clarify the level of human involvement, including care providers, health professionals, and technical assistance, in the e-intervention or as a co-intervention. Detail number and expertise of professionals involved and the type, timing, frequency, initiation, and medium of assistance. Distinguish the level of human involvement required for the trial from that required in routine application outside an RCT setting.	*Highly Recommended*	**pp 2, 11–13, and 28–29**
			xi) Report any prompts/reminders used: Clarify whether there were prompts such as letters, emails, phone calls, or SMS to use the application, what triggered them, and their frequency. Distinguish prompts/reminders required for the trial from those for routine application outside an RCT setting.	*Essential*	**Not used; see p 7**
			xii) Describe any co-interventions, including training/support: Clearly state any interventions provided in addition to the targeted eHealth intervention, including training sessions and support. Distinguish the level of training required for the trial from the level of training for routine application outside an RCT setting.	*Essential*	**pp 11–13 and 28–29**
Outcomes	6a	Completely defined pre-specified primary and secondary outcome measures, including how and when they were assessed	i) If outcomes were obtained through online questionnaires, describe whether they were validated for online use and apply CHERRIES items to describe how the questionnaires were designed/deployed.	*Highly Recommended*	**Not applicable; see pp 7 and 15–16**
			ii) Describe whether and how use, including intensity of use/dosage, was defined, measured, or monitored, e.g., logins or logfile analysis. Use/adoption metrics are important process outcomes that should be reported in any eHealth trial.	*Highly Recommended*	**pp 11–12 and 20**

Section/Topic	Item No.	CONSORT* Checklist Item	EHEALTH Extensions (additions to, or clarification of the CONSORT item)	Importance	Reported on page No
			iii) Describe whether, how, and when qualitative feedback was obtained from participants, e.g., through emails, feedback forms, interviews, or focus groups.	*Highly Recommended*	**Not collected as a study outcome; prespecified outcomes and outcome data sources are reported on pp 15–16.**
	6b	Any changes to trial outcomes after the trial commenced, with reasons	No EHEALTH-specific additions here.		**p 7**
Sample size	7a	How sample size was determined<br>NPT: When applicable, details of whether and how clustering by care providers or centers was addressed	i) Describe whether and how expected attrition was taken into account when calculating the sample size.	*Highly Recommended*	**pp 16–17**
	7b	When applicable, explanation of any interim analyses and stopping guidelines	No EHEALTH-specific additions here.		**p 17**
Randomisation: Sequence generation	8a	Method used to generate the random allocation sequence<br>NPT: When applicable, how care providers were allocated to each trial group	No EHEALTH-specific additions here.		**pp 9–10**
	8b	Type of randomisation; details of any restriction (such as blocking and block size)	No EHEALTH-specific additions here.		**p 9**
Allocation concealment mechanism	9	Mechanism used to implement the random allocation sequence (such as sequentially numbered containers), describing any steps taken to conceal the sequence until interventions were assigned	No EHEALTH-specific additions here.		**pp 9–10**
Implementation	10	Who generated the random allocation sequence, who enrolled participants, and who assigned participants to interventions	No EHEALTH-specific additions here.		**pp 9–10; the allocation sequence was generated and kept by an investigator independent of recruitment, training, clinical supervision, and outcome assessment.**
Blinding	11a	If done, who was blinded after assignment to interventions (for example, participants, care providers, those assessing outcomes) and how<br>NPT: Whether or not administering co-interventions were blinded to group assignment	i) Specify who was blinded and who was not. Usually, in web-based trials it is not possible to blind participants; this should be clearly acknowledged. It may be possible to blind outcome assessors, data analysts, or those administering co-interventions.	*Essential*	**pp 10–11, 14–15, and 28–29**

Section/Topic	Item No.	CONSORT* Checklist Item	EHEALTH Extensions (additions to, or clarification of the CONSORT item)	Importance	Reported on page No
			ii) Informed consent procedures can create biases and certain expectations. Discuss, for example, whether participants knew which intervention was the intervention of interest and which one was the comparator.	*Highly Recommended*	**pp 7–8 and 10–11; written informed consent was obtained offline, patients were not informed about the trainees' training modality, and trainees/instructors could not be blinded because the interventions were visually and operationally distinct.**
Blinding	11b	If relevant, description of the similarity of interventions	This item is usually not relevant for eHealth trials, as it refers to similarity of a placebo or sham intervention to an active medication/intervention.		**Not applicable; no placebo or sham intervention was used, and the distinct training modalities/non-blinding are reported on pp 10–11.**
Statistical methods	12a	Statistical methods used to compare groups for primary and secondary outcomes<br>NPT: When applicable, details of whether and how clustering by care providers or centers was addressed	i) Imputation techniques to deal with attrition/missing values: Not all participants will use the intervention/comparator as intended and attrition is typically high in eHealth trials. Specify how participants who did not use the application or dropped out were treated in the statistical analysis. Complete case analysis is strongly discouraged, and simple imputation techniques such as LOCF may also be problematic.	*Essential*	**pp 17–19**
	12b	Methods for additional analyses, such as subgroup analyses and adjusted analyses	No EHEALTH-specific additions here.		**p 19**
Ethics & Informed Consent	X26	(not a CONSORT item)	i) Comment on ethics committee approval.	*Highly Recommended*	**pp 7–8 and 30**
			ii) Outline informed consent procedures, e.g., whether consent was obtained offline or online, how it was obtained (checkbox, etc.), and what information was provided.	*Highly Recommended*	**pp 7–8 and 30**
			iii) Safety and security procedures, including privacy considerations and any steps taken to reduce the likelihood or detection of harm, e.g., education and training or availability of a hotline.	*Highly Recommended*	**pp 7–8, 15–16, 22, and 30**
**RESULTS**					
Participant flow (a diagram is strongly recommended)	13a	For each group, the numbers of participants who were randomly assigned, received intended treatment, and were analysed for the primary outcome<br>NPT: Number of care providers or centers performing the intervention in each group and number of patients treated by each care provider in each center	No EHEALTH-specific additions here.		**pp 20 and 38**

Section/Topic	Item No.	CONSORT* Checklist Item	EHEALTH Extensions (additions to, or clarification of the CONSORT item)	Importance	Reported on page No
	13b	For each group, losses and exclusions after randomisation, together with reasons	i) Strongly recommended: An attrition diagram, e.g., proportion of participants still logging in or using the intervention/comparator in each group plotted over time, similar to a survival curve, or other figures/tables demonstrating usage, dose, or engagement.	*Highly Recommended*	**pp 20 and 38**
Recruitment	14a	Dates defining the periods of recruitment and follow-up	i) Indicate if critical secular events fell into the study period, e.g., significant changes in Internet resources available or changes in computer hardware or Internet delivery resources.	*Highly Recommended*	**pp 6–7, 12, 16, and 22**
	14b	Why the trial ended or was stopped early	No EHEALTH-specific additions here.		**pp 7 and 17**
Baseline data	15	A table showing baseline demographic and clinical characteristics for each group NPT: When applicable, a description of care providers (case volume, qualification, expertise, etc.) and centers (volume) in each group	i) In eHealth trials it is particularly important to report demographics associated with digital divide issues, such as age, education, gender, socioeconomic status, and computer/Internet/eHealth literacy of participants, if known.	*Essential*	**pp 20 and 40**
Numbers analysed	16	For each group, number of participants (denominator) included in each analysis and whether the analysis was by original assigned groups	i) Report multiple denominators and provide definitions. Report Ns and effect sizes across a range of study participation and use thresholds, e.g., N exposed, N consented, N used more than x times, N used more than y weeks, and N participants who used the intervention/comparator at specific predefined time points of interest. Always clearly define use of the intervention.	*Essential*	**pp 11–12 and 20**
			ii) Primary analysis should be intent-to-treat; secondary analyses could include comparing only users, with the caveat that this is no longer a randomized sample.	*Highly Recommended*	**p 17**
Outcomes and estimation	17a	For each primary and secondary outcome, results for each group, and the estimated effect size and its precision (such as 95% confidence interval)	i) In addition to primary/secondary clinical outcomes, presentation of process outcomes such as metrics of use and intensity of use (dose, exposure) and their operational definitions is critical. This includes metrics of attrition and continuous exposure metrics such as average session length, accompanied by a technical description of how metrics such as a session are defined, e.g., timeout after idle time.	*Highly Recommended*	**pp 11–12, 20–22, and 41–44**
	17b	For binary outcomes, presentation of both absolute and relative effect sizes is recommended	No EHEALTH-specific additions here.		**pp 21 and 43**
Ancillary analyses	18	Results of any other analyses performed, including subgroup analyses and adjusted analyses, distinguishing pre-specified from exploratory	i) A subgroup analysis comparing only users is not uncommon in eHealth trials, but if done it must be stressed that this is a self-selected sample and no longer an unbiased sample from a randomized trial.	*Highly Recommended*	**p 19**
Harms	19	All important harms or unintended effects in each group (for specific guidance see CONSORT for harms)	i) Include privacy breaches and technical problems. This does not only include physical harm to participants, but also incidents such as perceived or real privacy breaches, technical problems, and other unexpected/unintended incidents. Unintended effects also include unintended positive effects.	*Highly Recommended*	**pp 15–16 and 22**
			ii) Include qualitative feedback from participants or observations from staff/researchers, if available, on strengths and shortcomings of the application, especially if they point to unintended/unexpected effects or uses. This includes, if available, reasons why people did or did not use the application as intended by the developers.	*Highly Recommended*	**Not collected; harms and unintended technical/privacy events were assessed and reported on pp 15–16 and 22.**
			**DISCUSSION**		
Limitations	20	Trial limitations, addressing sources of potential bias, imprecision, and, if relevant, multiplicity of analyses	i) Typical limitations in eHealth trials: Participants in eHealth trials are rarely blinded. eHealth trials often look at a multiplicity of outcomes, increasing risk for a Type I error. Discuss biases due to non-use of the intervention/usability issues, biases through informed consent procedures, and unexpected events.	*Essential*	**pp 26–29**
Generalisability	21	Generalisability (external validity, applicability) of the trial findings NPT: External validity of trial findings according to the intervention, comparators, patients, and care providers or centers involved in the trial	i) Generalizability to other populations: In particular, discuss generalizability to a general Internet population, outside of an RCT setting, and to the general patient population, including applicability of the study results for other organizations.	*Highly Recommended*	**pp 28–29**
			ii) Discuss if there were elements in the RCT that would be different in a routine application setting, e.g., prompts/reminders, more human involvement, training sessions or other co-interventions, and what impact omission of these elements could have on use, adoption, or outcomes outside an RCT setting.	*Highly Recommended*	**pp 25 and 28–29**
Interpretation/Principal Findings	22	Interpretation consistent with results, balancing benefits and harms, and	i) Restate study questions and summarize the answers suggested by the data, starting with primary outcomes and process outcomes (use).	*Essential*	**pp 23–25**

Section/Topic	Item No.	CONSORT* Checklist Item	EHEALTH Extensions (additions to, or clarification of the CONSORT item)	Importance	Reported on page No
		considering other relevant evidence<br>NPT: In addition, take into account the choice of comparator, lack of or partial blinding, and unequal expertise of care providers or centers in each group	ii) Highlight unanswered new questions and suggest future research.	*Highly Recommended*	**pp 26 and 29**
**OTHER INFORMATION**					
Registration	23	Registration number and name of trial registry	No EHEALTH-specific additions here.		**pp 3 and 6–7**
Protocol	24	Where the full trial protocol can be accessed, if available	No EHEALTH-specific additions here.		**Protocol details were finalized before participant screening and remained unchanged; see p 7. The full trial protocol was not publicly available.**
Funding	25	Sources of funding and other support (such as supply of drugs), role of funders	No EHEALTH-specific additions here.		**p 31**
Competing interests	X27	(not a CONSORT item)	i) In addition to the usual declaration of interests, financial or otherwise, also state the relation of the study team towards the system being evaluated, i.e., state if the authors/evaluators are distinct from or identical with the developers/sponsors of the intervention.	*Highly Recommended*	**pp 30–31**

* CONSORT = Consolidated Standards of Reporting Trials. ** NPT = non-pharmacological treatment (CONSORT extension).

Source: CONSORT-EHEALTH checklist (V.1.6.1). The final column is intentionally left blank for manuscript page numbers.
