# Supplementary material for: Haptic Virtual Reality Training for Combined Spinal-Epidural Anesthesia in Anesthesiology Trainees: Prospective Randomized Controlled Pilot Trial
Source: JMIR Serious Games. 2026 Oct 8;14:e92207. doi: 10.2196/92207 (PMC13649171; doi:10.2196/92207)
Supplement: Supplementary file 3 [file games-v14-e92207-s001.docx]

Modified Spinal-Epidural Puncture Assessment Scale used to evaluate clinical performance during supervised combined spinal-epidural anesthesia procedures in novice anesthesiology trainees in a single-center, prospective, parallel-group randomized pilot trial at Fujian Provincial Hospital, Fuzhou, China, March 2021.

| **Puncture Step** | **Score** | **Points awarded** |
| --- | --- | --- |
| 1. Proper patient positioning and identification of the puncture interspace | 8 |  |
| 2. Range and sequence of disinfection | 5 |  |
| 3. Draping | 3 |  |
| 4. Drawing up local anesthetic | 3 |  |
| 5. Local infiltration anesthesia | 3 |  |
| 6. Skin puncture | 3 |  |
| 7. Vertical insertion of the epidural puncture needle | 10 |  |
| 8. Bubble compression test using a mixture of air and saline to assess resistance | 10 |  |
| 9. Feeling of “loss of resistance” upon breakthrough of the ligamentum flavum | 10 |  |
| 10. Needle entry into the epidural space | 10 |  |
| 11. Avoidance of arachnoid membrane puncture | 10 |  |
| 12. Insertion of spinal needle, feeling of breakthrough, removal of stylet, and observation of cerebrospinal fluid flow | 3 |  |
| 13. Injection of local anesthetic (e.g., ropivacaine) diluted with cerebrospinal fluid | 3 |  |
| 14. Withdrawal of spinal needle | 3 |  |
| 15. Insertion of epidural catheter and confirmation of no blood or cerebrospinal fluid on aspiration | 3 |  |
| 16. Securing the catheter | 3 |  |
| 17. Compliance with aseptic technique | 10 |  |

Scoring guidance: Each item had a predefined maximum score. Raters assigned scores according to the degree of completion, technical correctness, procedural sequence, and patient safety of each step. Full points were awarded when the step was performed correctly, completely, and safely without prompting or correction. Partial scores, including half-point increments, were allowed when performance partially met the criteria or showed minor technical deficiencies. Zero points were assigned when the step was omitted, performed incorrectly, or required major correction for safety. The same proportional scoring principle was applied across items with different maximum scores. The final analyzed score for each item or domain was calculated as the mean of the two independent blinded raters' scores. Total score: 100 points.

**General scoring anchors for items with different maximum scores**

| **Maximum score** | **Full performance** | **Minor deficiency** | **Partial performance** | **Major deficiency** | **Omitted**  **/unsafe** |
| --- | --- | --- | --- | --- | --- |
| 10 points | 9-10 | 7-8 | 5-6 | 1-4 | 0 |
| 8 points | 7-8 | 5-6 | 3-4 | 1-2 | 0 |
| 5 points | 5 | 4 | 2-3 | 1 | 0 |
| 3 points | 3 | 2 | 1 | 0.5 | 0 |
