# Supplementary material for: Haptic Virtual Reality Training for Combined Spinal-Epidural Anesthesia in Anesthesiology Trainees: Prospective Randomized Controlled Pilot Trial
Source: JMIR Serious Games. 2026 Oct 8;14:e92207. doi: 10.2196/92207 (PMC13649171; doi:10.2196/92207)
Supplement: Supplementary file 4 [file games-v14-e92207-s002.docx]

Baseline characteristics of patients undergoing supervised combined spinal-epidural anesthesia procedures performed by anesthesiology trainees in a single-center, prospective, parallel-group randomized pilot trial comparing haptic virtual reality–based training vs traditional manikin-based training at Fujian Provincial Hospital, Fuzhou, China, March 2021.

| Parameter | CG (n=10) | SG (n=10) | *P* |
| --- | --- | --- | --- |
| Age (years) | 56.60 (5.25) | 59.90 (6.15) | 0.21 |
| Sex (M/F) | 4/6 | 5/5 | 0.65 |
| BMI (kg/m²) | 21.22 (1.23) | 20.61 (1.02) | 0.24 |
| ASA classification (I/II) | 5/5 | 3/7 | 0.36 |

M: male; F: female; BMI: body mass index; ASA: American Society of Anesthesiologists; CG: control group; SG: simulator group.
